# Dataset on investigating an optimal household waste management in GIS environment and quantitative and qualitative analysis in Bumehen city, Tehran, Iran

**DOI:** 10.1016/j.dib.2018.08.008

**Published:** 2018-08-09

**Authors:** Mohammad Hadi Dehghani, Aida Biati, Zahra Mirzaeian, Zoha Heidarinejad

**Affiliations:** a Department of Environmental Health Engineering, School of Public Health, Tehran University of Medical Sciences, Tehran, Iran; b Institute for Environmental research, Center for Solid Waste Research, Tehran University of Medical Sciences, Tehran, Iran; c Islamic Azad University, Science and Research Branch, Faculty of environment & energy, Department of environmental engineering, Tehran, Iran; d Department of Environmental Health Engineering, Faculty of Health, Hormozgan University of Medical Sciences, Bandar Abbas, Iran

**Keywords:** Household waste, Storage, Collection, Transfer, GIS, Bumehen

## Abstract

The present data was carried out based on macro policies of the municipality in order to optimize the waste collection and transportation system in the city of Bumehen. The data of this research, the average weights, time taken for collection and transportation of municipal solid waste (MSW) was measured and each of these criteria was calculated and evaluated from environmental and time perspectives under the current management system. Then, data collection, identification of the general characteristics of the region and the type of waste management were conducted using field studies and GIS software was later used to generate maps of classes, route type, per capita waste generation. In the next steps and based on information such as density, population, waste generation capacity, available routes and existing route types, number, type and capacity of tanks, the site of temporary transfer stations was determined and the appropriate routes were designed for the garbage trucks. The data showed that distance from urban space, as a physical criterion, and noise pollution, as an environmental criterion, in the selection of urban waste transfer station of a relative weight of 0.594 is the most important indicator for the construction of a waste transfer station. Also, the qualitative analysis of the dry wastes of the city of Bumehen showed that plastics, cardboard and paper were 8.6%, 8.6% and 8.3% respectively, of the highest amount of waste the city Bumehen formed.

**Specifications Table**

**Table:** 

Subject area	Environmental Health
More specific subject area	Waste Management
Type of data	Table, Figures
How data was acquired	In the first stage, observations and studies on the status of the existing area were investigated for texture and waste management. In the second stage, the physical analysis of waste was carried out in the city of Bumehen. In the third stage, the design of the optimal system was performed using Arc GIS. V 10.3 software.
Data format	Raw, Analyzed
Experimental factors	1. Check the status of waste management Bumehen city, Tehran 2. The optimal storage system for collecting and transporting waste was designed with Arc GIS software. 3. Analytical Hierarchy Process (AHP) method was used to provide a solution.
Experimental features	The purpose of this data was to: - Design the transport routes and storage system; - Provide suitable solutions for improving municipal waste management.
Data source location	Islamic Azad University, Tehran, Iran.
Data accessibility	The data are available with this article

**Value of the data**

• The data will be useful for designing an optimal MSW collection and transportation system.
• The data will help municipalities and municipal waste management organizations improve MSW management.
• The data can help identify the quantity and quality of waste products in the city of Bumehen, and will be useful for recycling waste produced by recycling companies.

## 1 Data

**Fig. 1:**
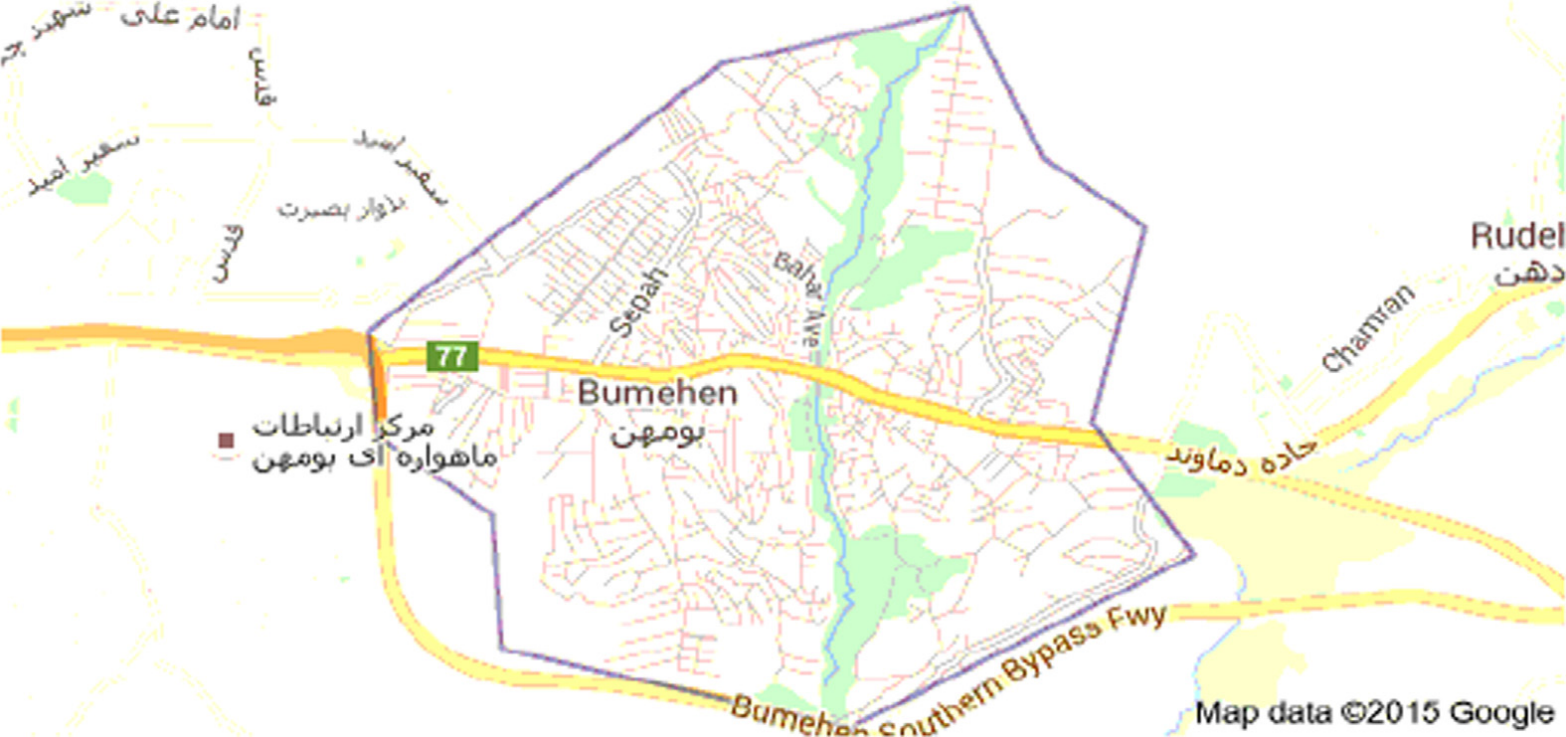
Location of the studied region, Bumehen city, Tehran, Iran.

**Fig. 2:**
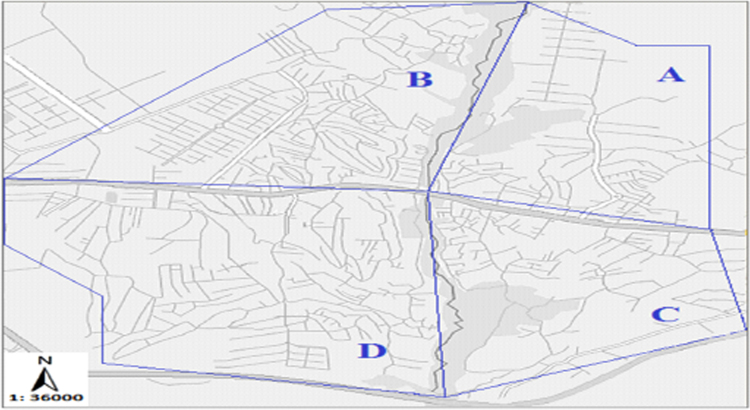
Four regions of the Bumehen Municipality: A: Region 1, B: Region 2, C: Region 3, D: Region 4.

**Fig. 3:**
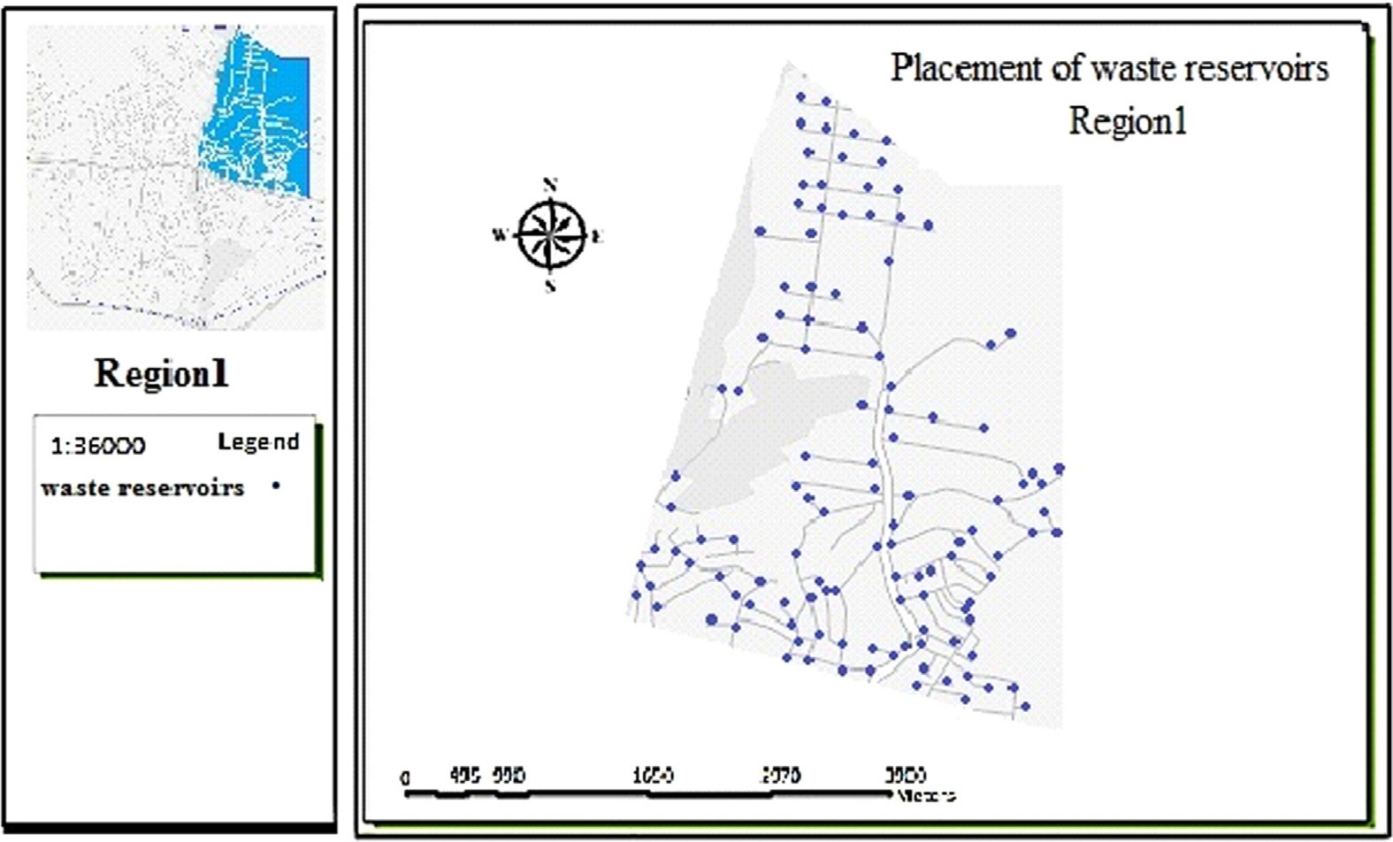
Optimal location of storage tanks in region 1.

**Fig. 4:**
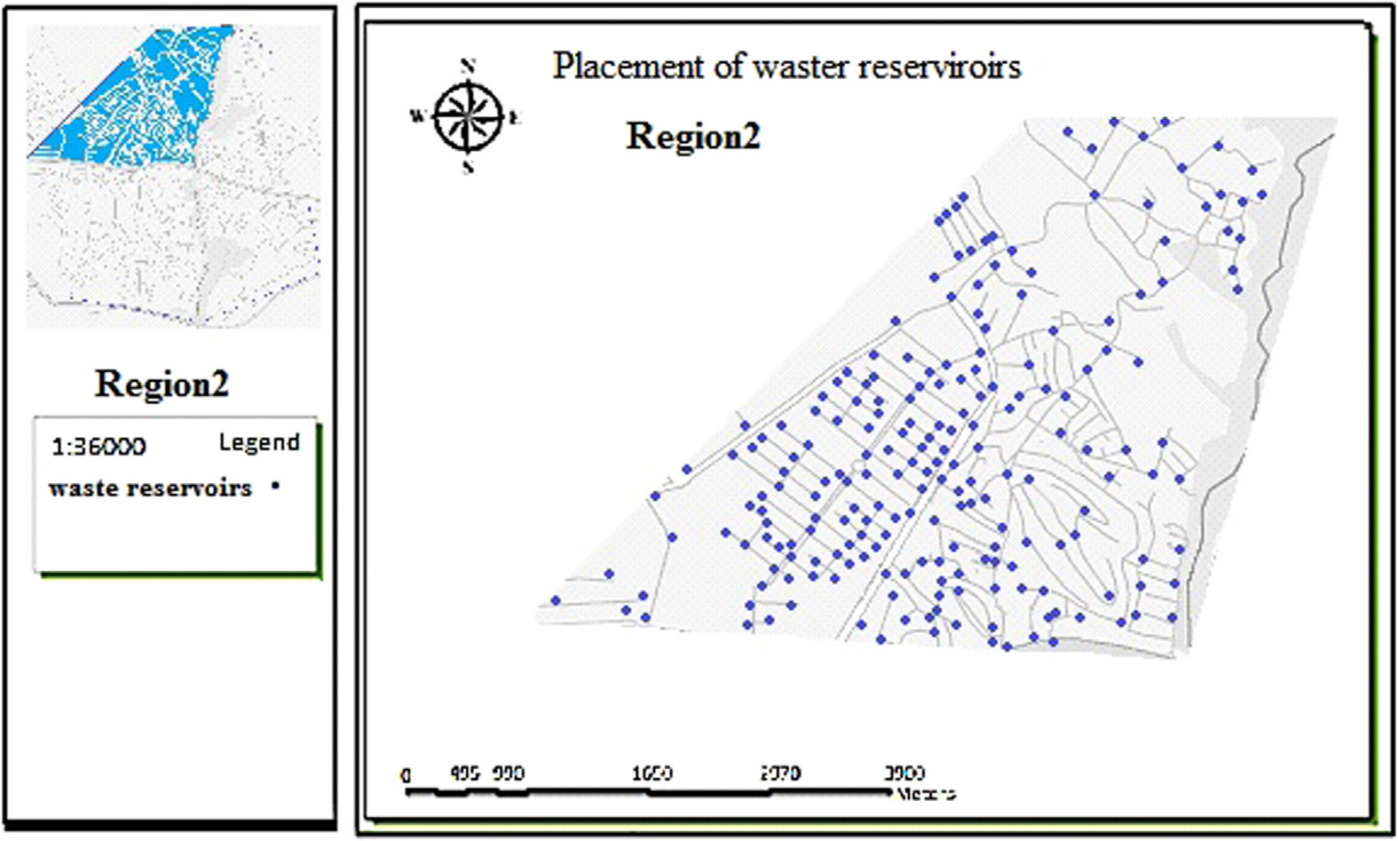
Optimal location of storage tanks in region 2.

**Fig. 5:**
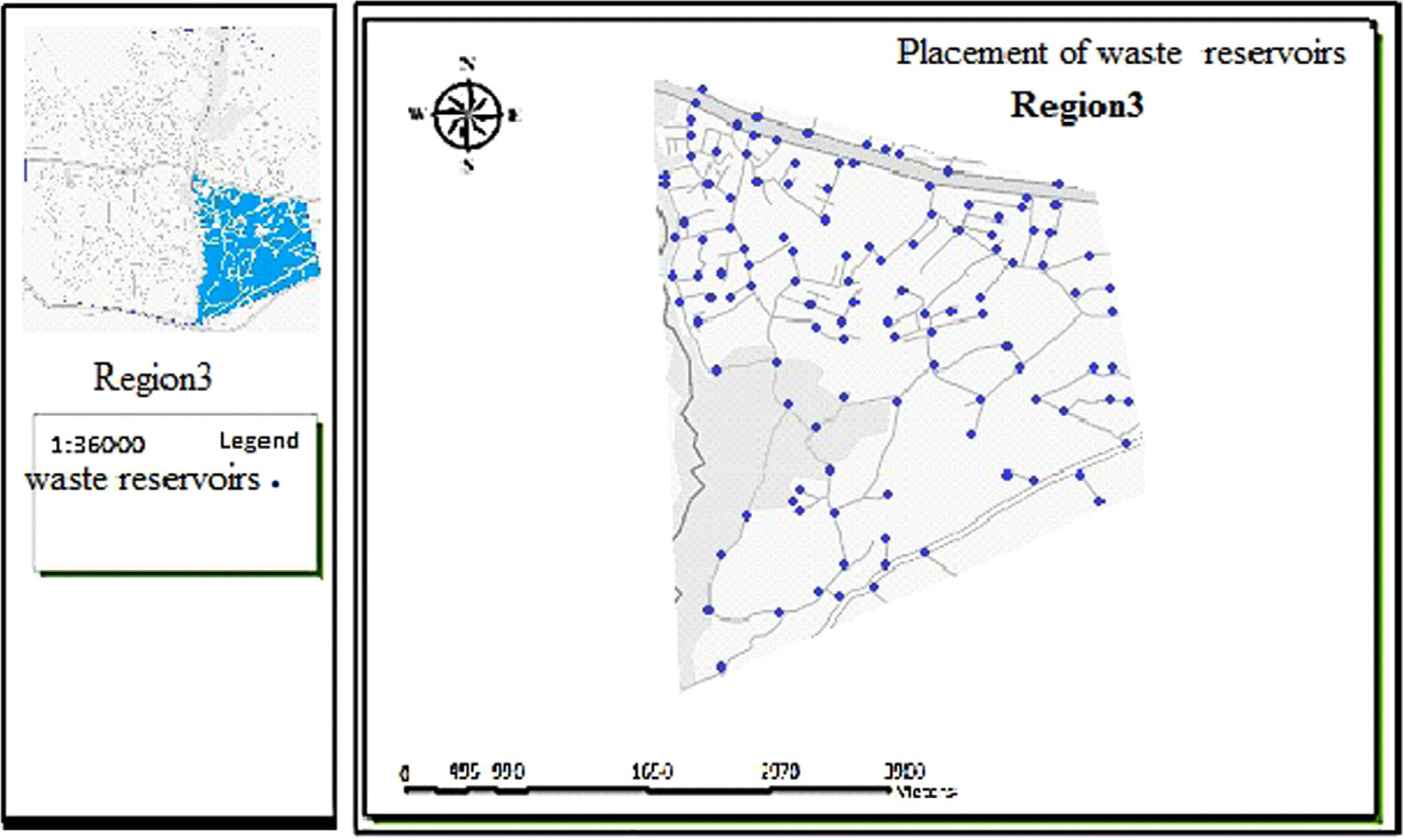
Optimal location of storage tanks in region 3.

**Fig. 6:**
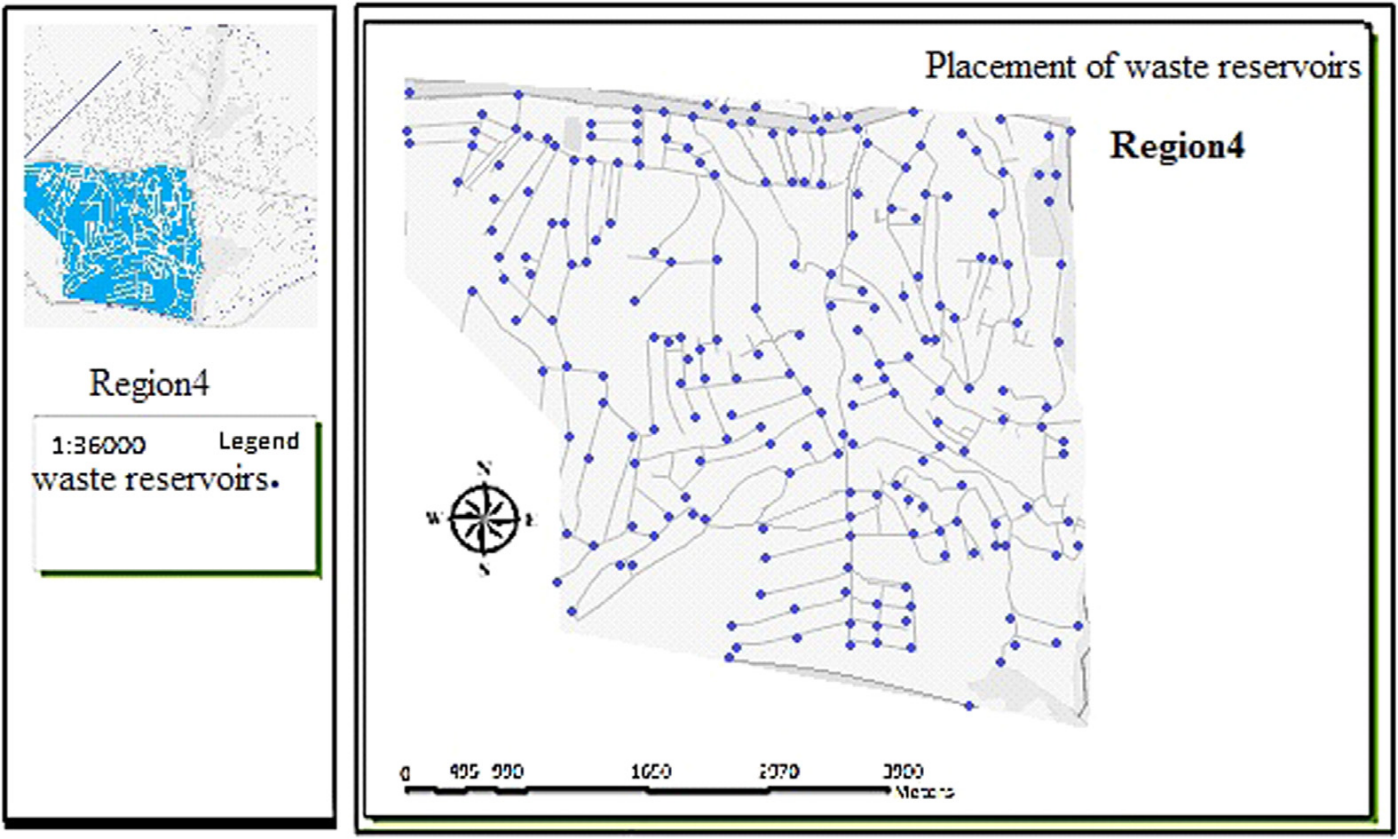
Optimal location of storage tanks in region 4.

**Fig. 7:**
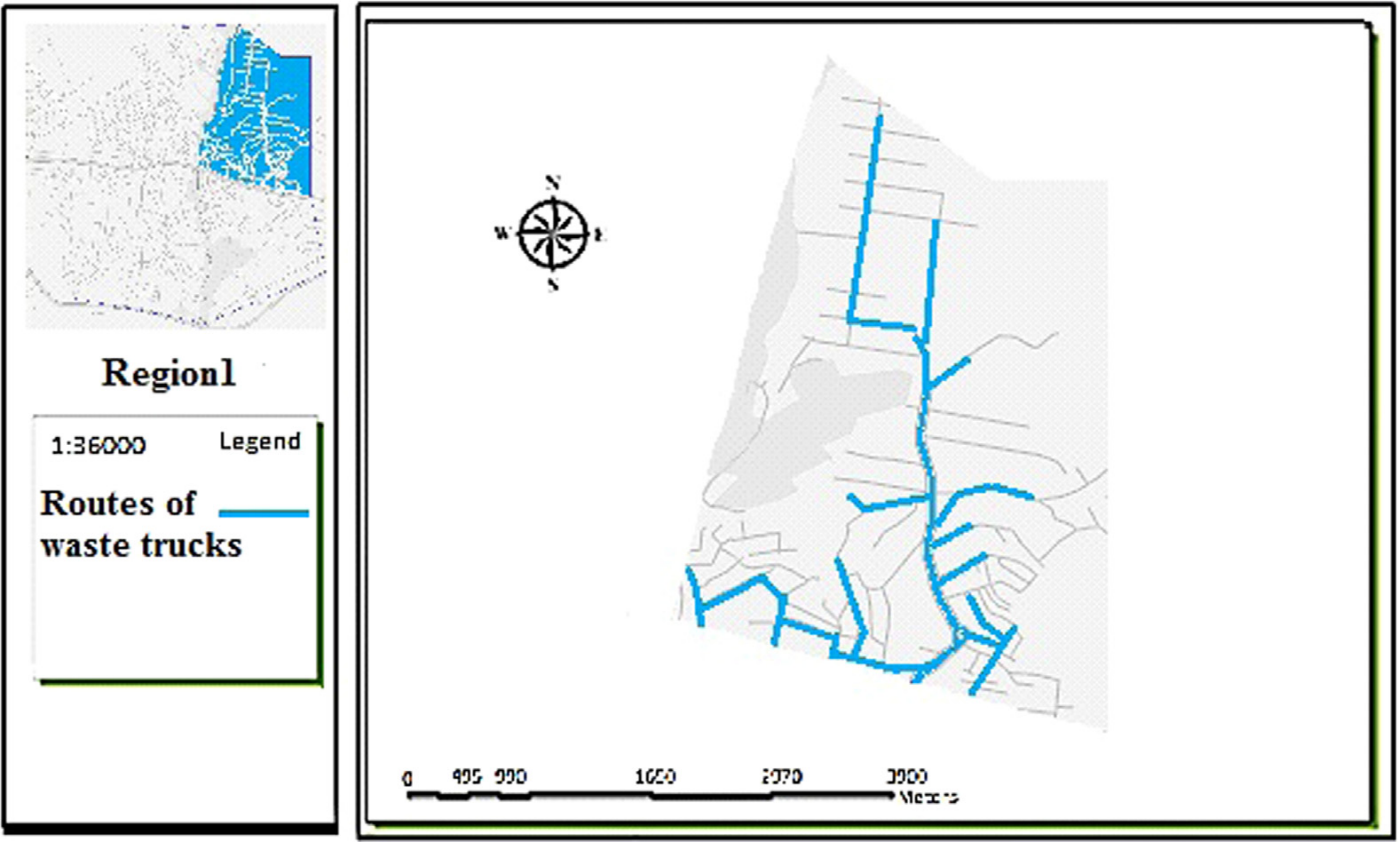
Waste transport routes in region 1.

**Fig. 8:**
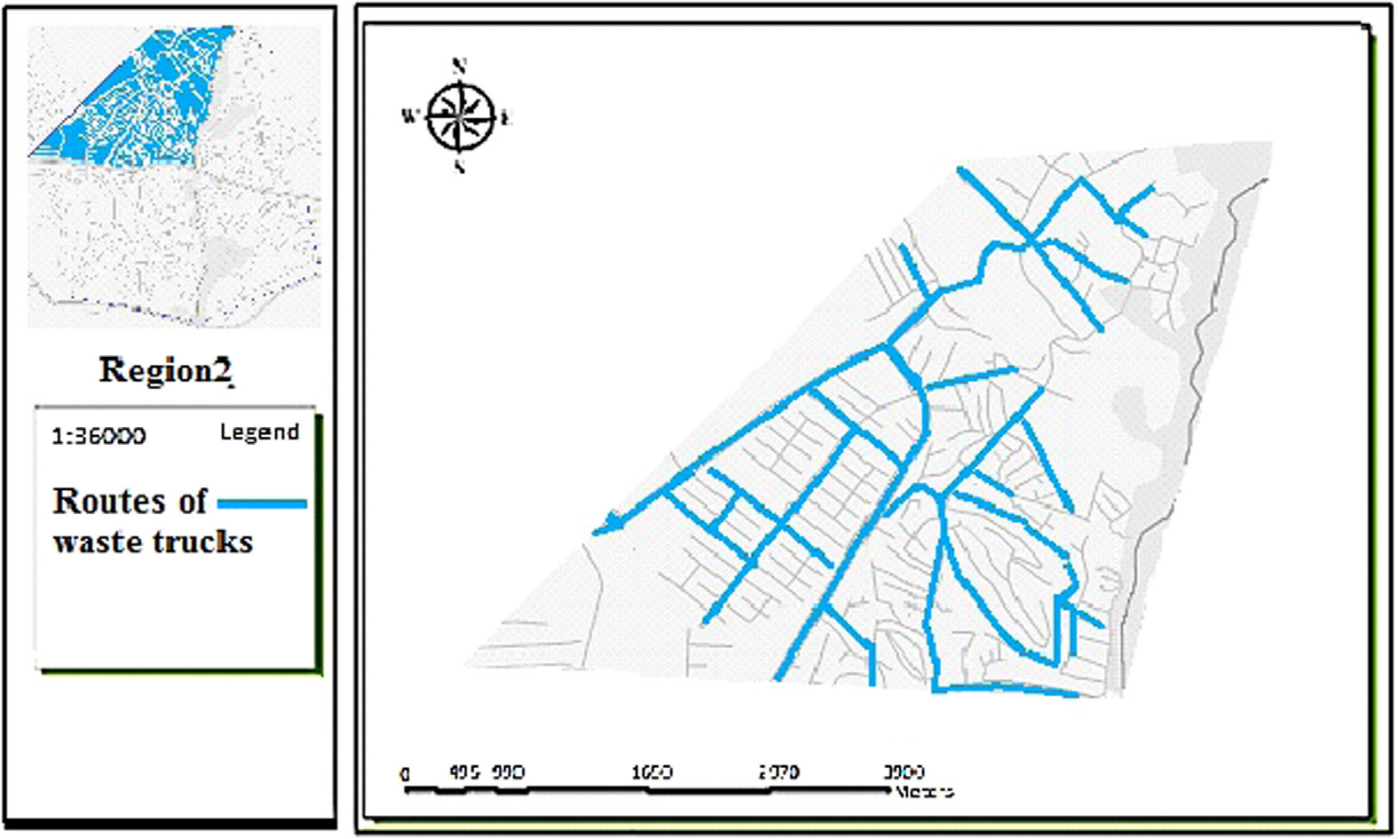
Waste transport routes in region 2.

**Fig. 9:**
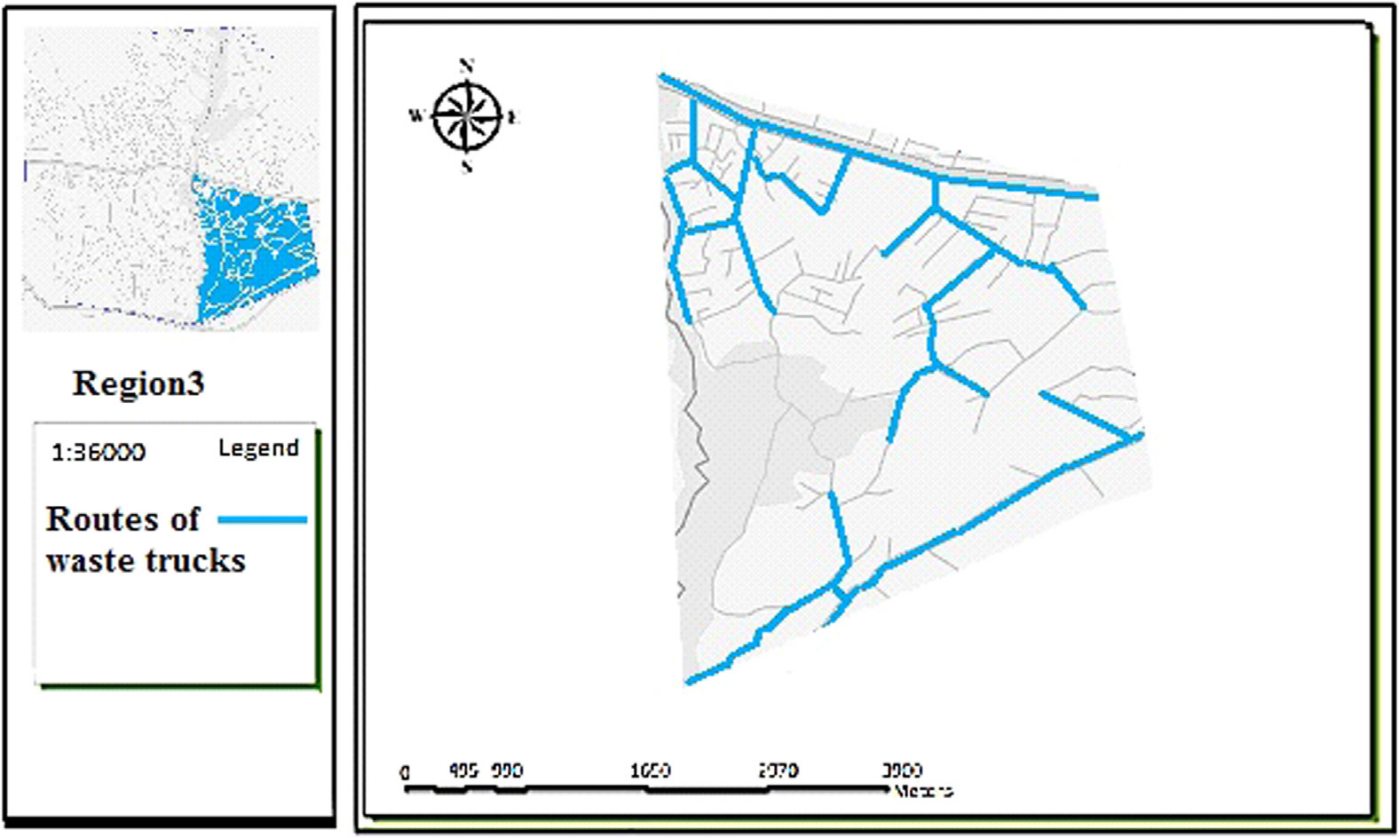
Waste transport routes in region 3.

**Fig. 10:**
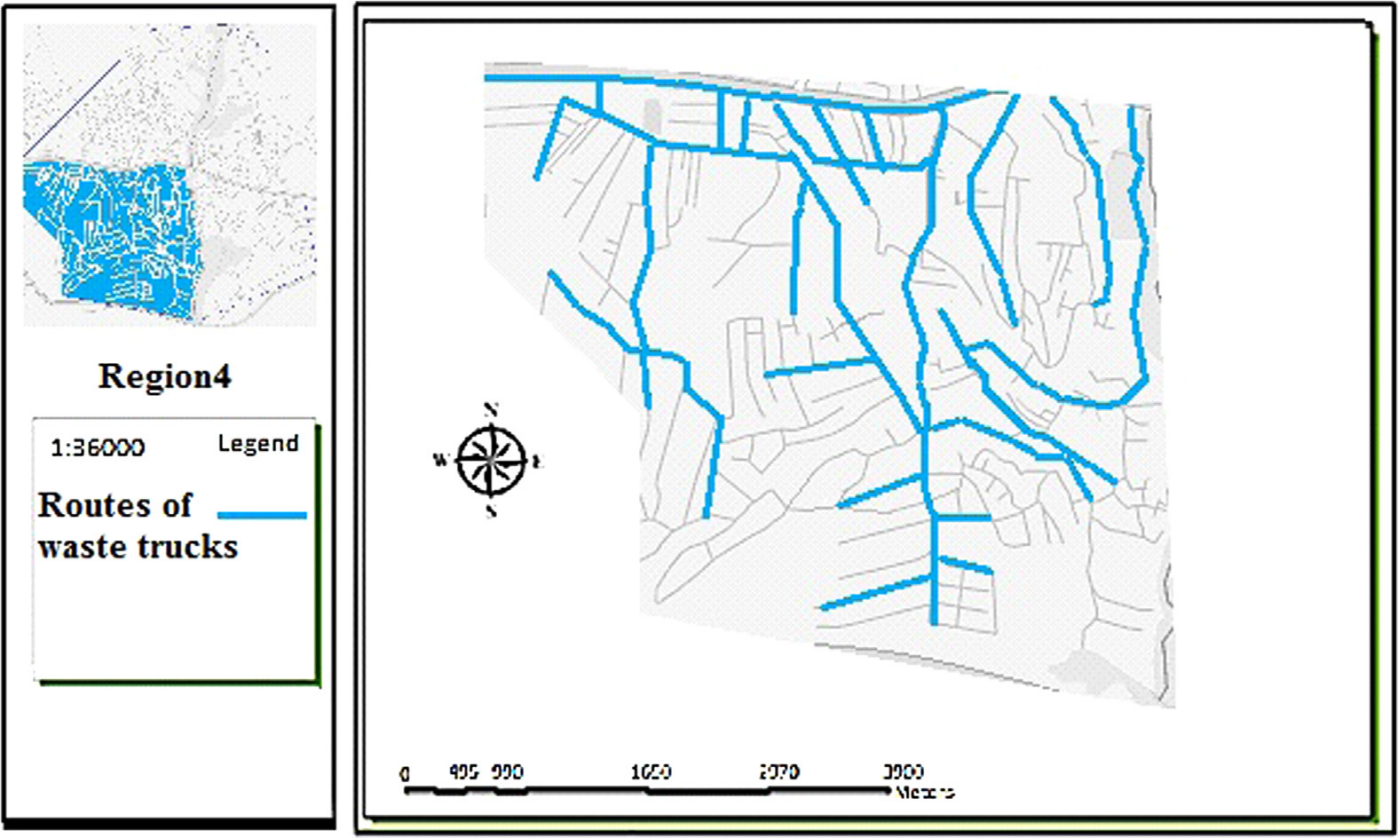
Waste transport routes in region 4.

**Fig. 11:**
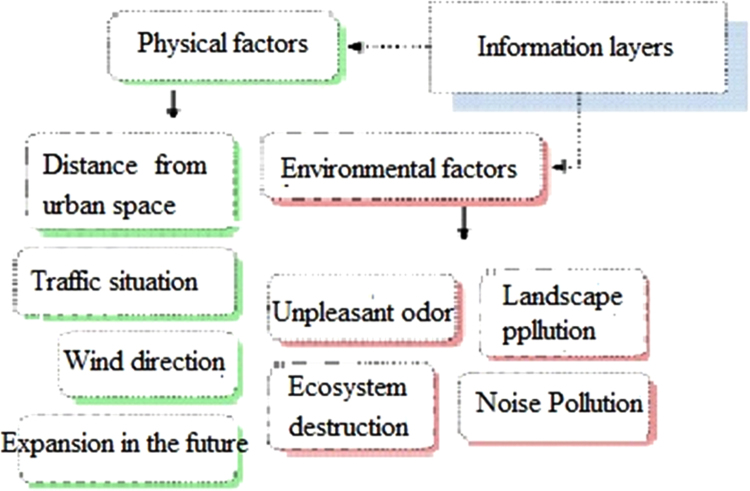
Hierarchical structure of criteria and sub-criteria.

**Fig. 12:**
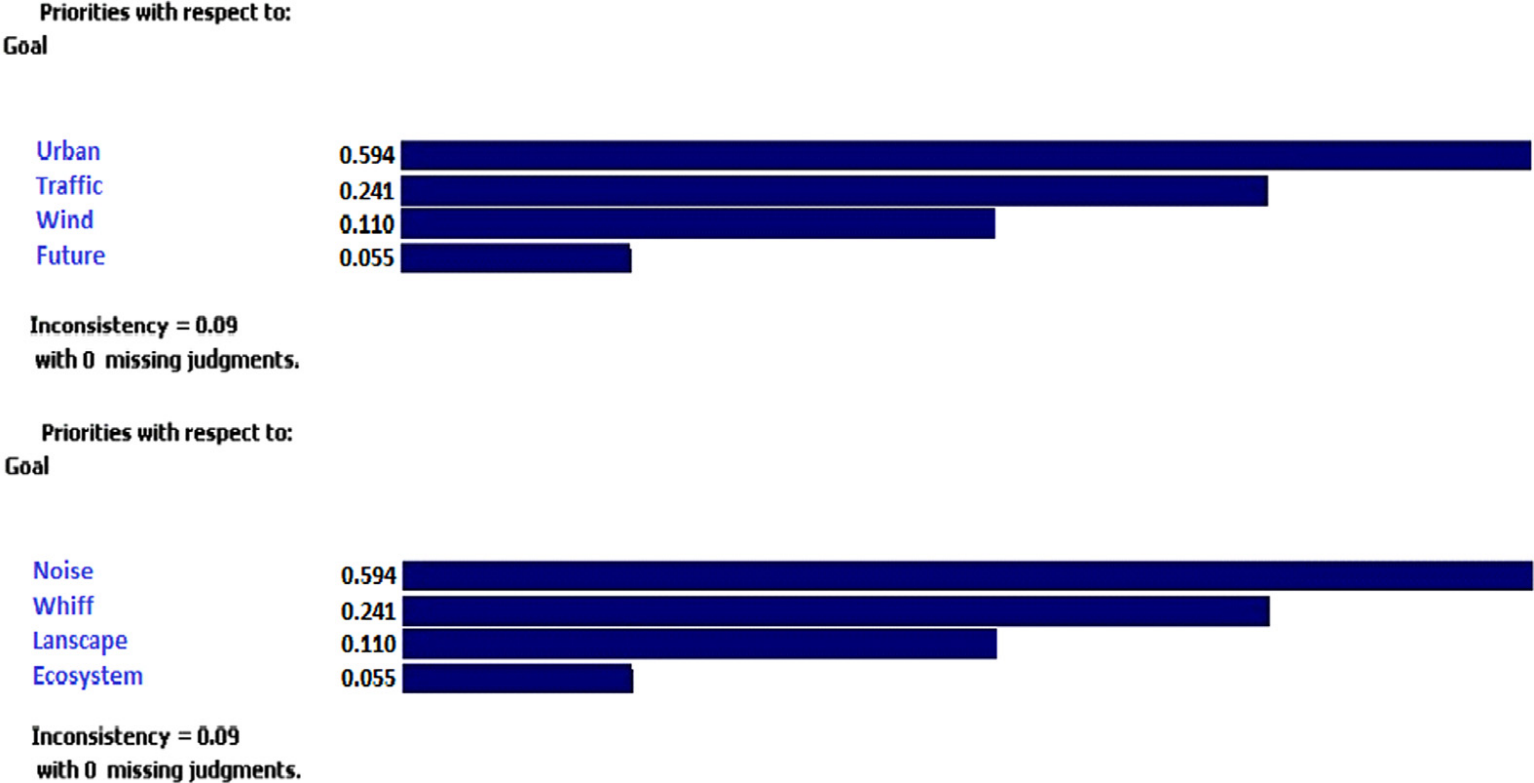
The results of the EC software physical (high) and environmental (low) sub-criteria.

**Fig. 13:**
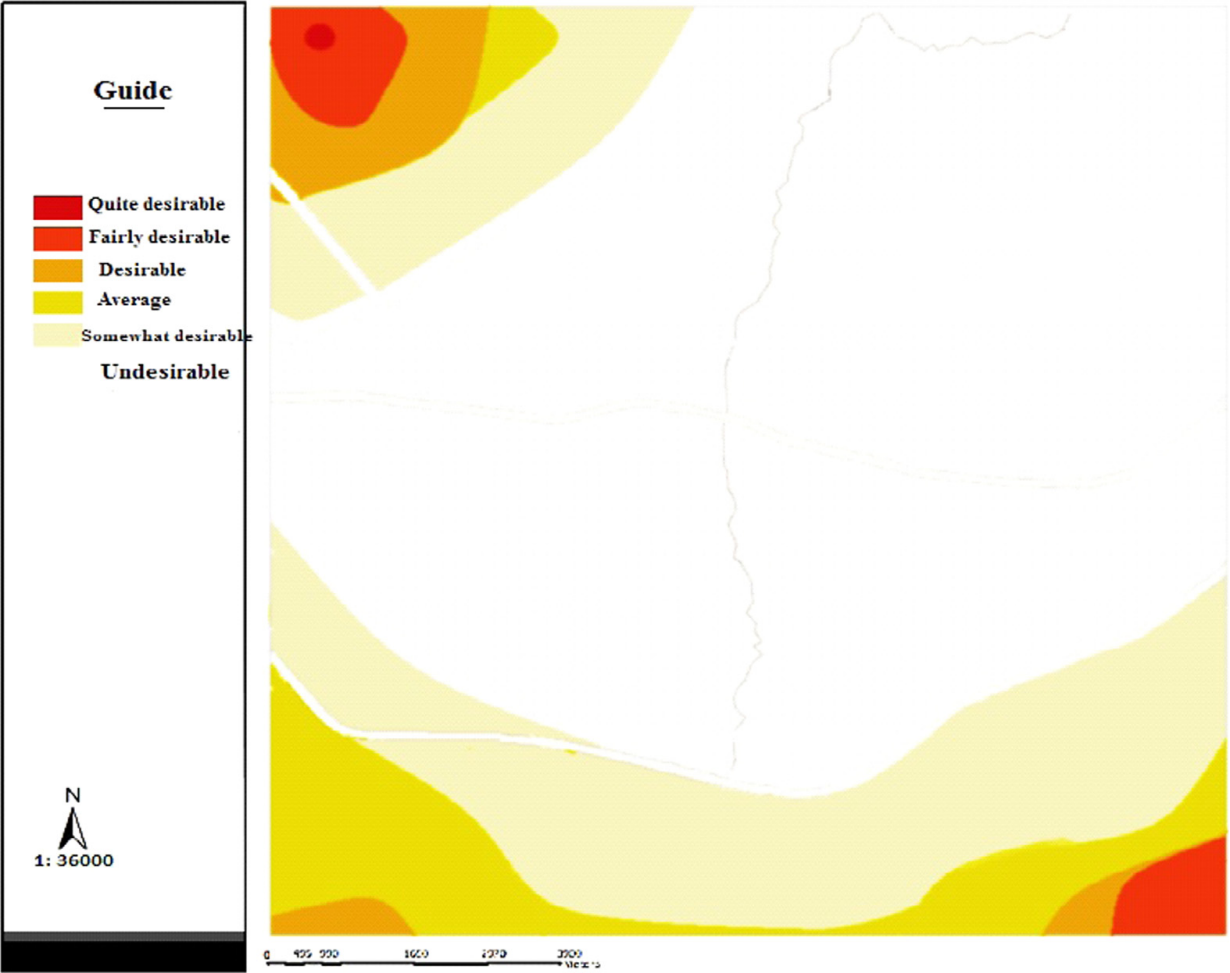
Prioritization of optimal location options for waste transfer station in the city of Bumehen.

The data includes 13 figures and 5 tables. The location of the studied region is shown in Fig. 1. Fig. 2 shows the four regions of the city of Bumehen. Table 1 shows the amount of waste generated in different regions and Table 2 shows the MSW qualitative analyses in the city of Bumehen. Table 3 shows MSW management status in different municipal regions of the city of Bumehen. Fig. 3, Fig. 4, Fig. 5, Fig. 6 show the optimal location of tanks in different parts of the city of Bumehen. Traffic routes for garbage trucks are shown in Fig. 7, Fig. 8, Fig. 9, Fig. 10. The final weight of the optimal selection options for transfer stations is shown in Table 5. The hierarchical structure of criteria and sub-criteria for determining the optimal waste storage system is presented in Fig. 11. Table 4 demonstrates indices weighting and comparison processes carried out using the AHP method and Fig. 12 illustrates the data of the EC software in determining the sub-criteria. The Fig. 13 shows prioritization of the optimal location options for the transfer station in Bumehen.

**Table 1:** The amount of waste generated by different regions of the city of Bumehen (quantitative analysis of MSW in the city of Bumehen).

Region name	Rate of waste generation (kg)	Population (person)	Per capita waste generation (g/person/day)
Region1	5980.9	8185	730.7
Region2	14,258.6	19,432	3733.7
Region3	3714.6	5016	740.5
Region4	15,319.7	20,818	735.8
Total	39,274	53,451	734.7

**Table 2:** Qualitative analysis of dry wastes of the city of bumehen.

Type	Ratio(Percent)	Cumulative percent	Relative to total waste (Percent)
Plastic	8.6	8.6	19.33
Cardboard	8.6	17.2	19.33
Paper	8.3	25.5	18.65
Colored metals	6.4	31.9	14.39
Dried bread	4.6	36.5	10.35
Glass	2.5	39	5.61
Ironware	1.7	40.7	3.82
Tires	1.1	41.8	2.47
Special waste	1.1	42.9	2.47
Cloth	0.6	43.5	1.34
Wood	0.4	43.9	0.90
Others (mica sheet, foam, etc.)	0.6	44.5	1.34
Total dry wastes	44.5		100

**Table 3:** Waste management status in different municipal regions of the city of Bumehen.

Region	Number of vehicles	Number of tank	Number of municipal	Population	Labor per capita
			solid waste workers	(person)	(per thousand)
1	2	86	4	8185	0.855
2	3	120	9	19,432	0.463
3	2	68	3	5016	0.59
4	3	138	9	20,818	0.432
Total	10	412	25	53,451	0.467

**Table 4:** Weighing indices and comparing them with each other using the AHP method.

**Index name**	**Noise**	**Unpleasant**	**Landscape**	**Ecosystem**	**Distance from**	**Traffic**	**Wind**	**Expansion**
	**Pollution**	**odor**	**destruction**	**destruction**	**urban space**	**situation**	**direction**	**in the future**
Noise pollution	1	2	3	4	5	7	8	9
Unpleasant odor	1.2	1	2	3	4	5	7	8
Landscape destruction	1.3	1.2	1	2	3	4	5	7
Ecosystem destruction	1.4	1.3	1.2	1	2	3	4	5
Distance from urban space	1.5	1.4	1.3	1.2	1	2	3	4
Traffic situation	1.7	1.5	1.4	1.3	1.2	1	2	3
Wind direction	1.8	1.7	1.5	1.4	1.3	1.2	1	2
Expansion in the future	1.9	1.8	1.7	1.5	1.4	1.3	1.2	1

**Table 5:** Final weight of the optimal selection options for the municipal waste transfer station.

Options / Criteria	1	2	3	Average
A	0.368	0.365	0.340	0.357
E	0.330	0.316	0.309	0.318
H	0.106	0.105	0.115	0.108
G	0.073	0.098	0.108	0.093
C	0.049	0.056	0.052	0.052
B	0.044	0.023	0.032	0.033
D	0.022	0.020	0.029	0.023
F	0.008	0.014	0.015	0.012

## 2 Experimental design, materials and methods

### 2.1 Geographical and climatic characteristics of the studied region

Bumehen is a city in the east of Tehran province, which is located 54 km Northeast to the left of the Tehran-Damavand road on the cold slopes. The city population is estimated to be 53,451 people (15,729 households) including 27,470 men and 25,981 women according to the 2011 national population and housing census. The location of the studied region is shown in Fig. 1. The Bumehen municipality organization has divided the city into four urban regions, as shown in Fig. 2.

### 2.2 Methods

Initially, basic information was prepared and collected through library studies, field operations, local visits, and interviews with experts working in the Urban Services Department of Bumehen. Then the physical waste analysis was carried out in Bumehen city and its waste management status was later determined. Finally, the optimal location of the temporary storage of waste and the traffic route of garbage trucks in the city of Bumehen were designed and identified using the Arc GIS.V 10.3 software and analytical hierarchy process (AHP) [1], [2], [3], [4], [5].

#### 2.2.1 The design of the waste collection system in the city of Bumehen

The collection system was designed based on population density, road width and accessibility, the shortest travel route from homes and per capita waste generation. The present state of the waste(s) collection tanks placement in the city׳s neighborhoods is completely traditional. However, as the experts stated, maps should be designed considering tanks spacing and road width of at least 100 and 4 m, respectively.

#### 2.2.2 Designing an optimal system for waste transport routes in the city of Bumehen

The waste transport routes were selected on their proximity to the main roads (the shortest); quicker access to the transfer site and the road leading to it, as well as only one-time passage through the road.

#### 2.2.3 Determining the optimal temporary waste storage system in Bumehen

Temporary waste storage stations will be built in order to reduce fuel consumption and subsequent pollution, as well as reducing other costs. Analytical Hierarchy Process (AHP) was used to extract, screen and select the location criteria of waste transfer stations [2]. Criteria and sub-criteria were selected after reviewing various information sources. These criteria were then presented to 15 experts working in the Urban Services and Recycling Department of Bumehen using Delphi Decision-Making Questionnaire in four stages. All of these experts hold education level of higher than BA (in the related field of study), and the criteria hierarchy was obtained, as shown in Fig. 11. After identifying the factors affecting the placement of the temporary waste storage station, their effect was evaluated using an appropriate tool. After entering the required data, the pairwise comparison matrix was calculated using Expert Choice software for criteria and sub-criteria in this research, and finally the relative weight of the above materials was obtained. Finally, the weight of each criterion was then calculated using EC software. Table 4 shows pairwise comparison matrix for the criteria. Then, weights of physical and environmental criteria and their sub-criteria were determined using the above software (Fig. 12). Also, the incompatibility coefficient of the related matrices was less than 0.1 for all of the above criteria, which indicated the desirable compatibility of all judgments. Below, we have discussed proposed standards based on the results of criteria prioritization as well as similar research records, considering the minimum requirements defined for each of the sub-criteria set in the optimal mode:

A. Environmental noise must be less than 55 dB per day, so it does not cause noise pollution; B. The transfer station should not be located in the direction of the dominant winds leading to residential areas;

C. The waste transfer must not eliminate the region natural landscape of each region;

D. The region ecosystem must not be affected by the location of the waste transfer station.

E. The transfer station must be located at a distance of at least 1000 m from urban space; F. The station must have enough space to expand the current use in the future; G. The unpleasant odor and air pollution caused by the transfer station should not affect the residential regions;

H. The traffic situation of the region should not be interrupted while the garbage trucks are moving
